# CD97 inhibits osteoclast differentiation via Rap1a/ERK pathway under compression

**DOI:** 10.1038/s41368-023-00272-x

**Published:** 2024-02-04

**Authors:** Wen Wang, Qian Wang, Shiying Sun, Pengfei Zhang, Yuyu Li, Weimin Lin, Qiwen Li, Xiao Zhang, Zhe Ma, Haiyan Lu

**Affiliations:** 1 Hebei Key Laboratory of Stomatology, Hebei Clinical Research Center for Oral Diseases, Hebei Medical University, Shijiazhuang, China; 2 Department of Orthodontics, School and Hospital of Stomatology, Hebei Medical University, Shijiazhuang, China; 3 State Key Laboratory of Oral Diseases & National Center for Stomatology & National Clinical Research Center for Oral Diseases & West China Hospital of Stomatology, Sichuan University, Chengdu, China; 4 Department of Preventive Dentistry, School and Hospital of Stomatology, Hebei Medical University, Shijiazhuang, Hebei China

**Keywords:** Orthodontics, Cell signalling

## Abstract

Acceleration of tooth movement during orthodontic treatment is challenging, with osteoclast-mediated bone resorption on the compressive side being the rate-limiting step. Recent studies have demonstrated that mechanoreceptors on the surface of monocytes/macrophages, especially adhesion G protein-coupled receptors (aGPCRs), play important roles in force sensing. However, its role in the regulation of osteoclast differentiation remains unclear. Herein, through single-cell analysis, we revealed that CD97, a novel mechanosensitive aGPCR, was expressed in macrophages. Compression upregulated CD97 expression and inhibited osteoclast differentiation; while knockdown of CD97 partially rescued osteoclast differentiation. It suggests that CD97 may be an important mechanosensitive receptor during osteoclast differentiation. RNA sequencing analysis showed that the Rap1a/ERK signalling pathway mediates the effects of CD97 on osteoclast differentiation under compression. Consistently, we clarified that administration of the Rap1a inhibitor GGTI298 increased osteoclast activity, thereby accelerating tooth movement. In conclusion, our results indicate that CD97 suppresses osteoclast differentiation through the Rap1a/ERK signalling pathway under orthodontic compressive force.

## Introduction

Malocclusion, defined as abnormal occlusion and/or disturbed craniofacial relationships, is one of three major oral diseases currently reported by the World Health Organization.^1^ It may impair the physical appearance of individuals, oral function, psychological health, and even quality of life.^2,3^

Orthodontic treatment is the main method of correcting malocclusion and obtaining coordinated relationships through orthodontic tooth movement (OTM).^4^ Periodontal tissue remodelling is a key biological process in OTM.^5^ However, this process is very slow. The long duration of orthodontic treatment greatly affects patient cooperation and satisfaction and may increase the incidence of adverse effects, such as enamel demineralisation and root resorption.^6,7^ Extensive surgical and nonsurgical strategies have been used to accelerate OTM.^8,9^ However, these methods have many limitations.^10^ Therefore, identifying more effective therapeutic targets is necessary for accelerating OTM.

OTM is a mechanobiological process based on the compression–tension theory.^11^ Bone resorption on the pressure side is the rate-limiting step, and osteoclast activation plays a decisive role in bone metabolism.^12^ Osteoclasts are derived from monocyte/macrophage haematopoietic precursors that terminally differentiate into mature osteoclasts.^13^ Under orthodontic force stimulation, a series of cytokines are released from periodontal fibroblasts and osteoblasts that indirectly regulate osteoclast differentiation.^14^ Recent studies have demonstrated that mechanical stimuli can directly regulate osteoclast differentiation through mechanosensitive receptors, including mechanosensitive ion channels and cell adhesion molecules.^15,16^ G protein-coupled receptors (GPCRs) are a large family of receptors located on the surface of cell membranes^17^ and are closely associated with osteoclast differentiation and maturation.^18,19^ Recent studies have shown that GPCRs can sense mechanical force and participate in intracellular biological processes.^20,21^ However, whether GPCRs participate in osteoclastogenesis by sensing mechanical forces is still unknown.

CD97, an adhesion G protein-coupled receptor (aGPCR), was first identified in haematopoietic cells.^22^ This molecule has well-documented effects on immune system diseases and epithelial cell-derived malignancies, but recently, the mechanosensory function of CD97 has attracted increased attention.^23^ Nevertheless, the expression of CD97 in periodontal tissue has not been reported. In addition, whether orthodontic forces alter CD97 expression remains unclear. Moreover, whether CD97 regulates osteoclast differentiation or is involved in OTM is not yet fully understood.

In the present study, we investigated the relationship between CD97 expression and osteoclast differentiation using models of OTM and static compression application. CD97 was identified as a potential mechanosensory that inhibited osteoclast differentiation and OTM. These findings will be helpful in understand the mechanism of OTM and hoping to provide a potential therapeutic target for accurately accelerating tooth movement.

## Results

### Single-cell atlas reveals the CD97 expression in monocytes-macrophages

**Fig. 1:**
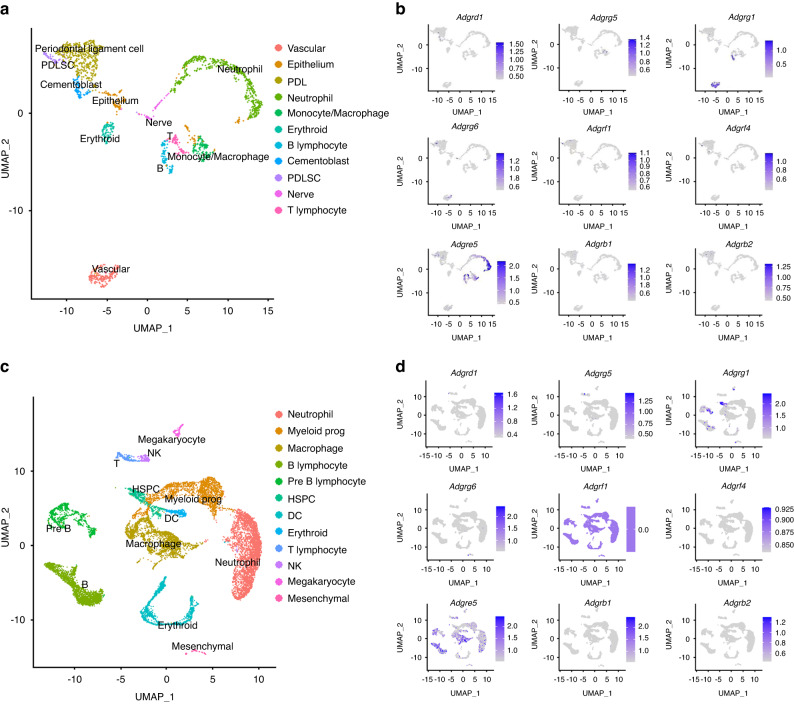
Single-cell atlas reveal the CD97 expression in monocytes-macrophages. **a** Cells from periodontal tissue was identified by scRNA-seq, and data were visualised with uniform manifold approximation and projection (UMAP). **b** A panel of aGPCR genes was projected onto UMAP atlas of periodontium. **c** Cells from alveolar bone was identified by scRNA-seq, and data were visualised with UMAP. **d** A panel of aGPCR genes was projected onto UMAP atlas of alveolar bone

We first analysed the published single-cell RNA sequencing (scRNA-seq) data from the periodontium and alveolar bone, respectively. Based on the classical marker genes, a total of 11 clusters from periodontal tissue were characterised: vascular—*Cdh5*, epithelium—*Krt14*, periodontal ligament cell (PDL)—*Dcn*, neutrophil—*Ly6g*, monocyte/macrophage—*Cd68*, erythroid cell—*Hbb-bt*, B lymphocyte—*Cd79a*, cementoblast—*Dmp1*, periodontal ligament stem cells (PDLSC)—*Cxcl12*, nerve—Plp1 and T lymphocyte—*Cd3g* (Fig. 1a and Supplementary Fig. 1a). Macrophage subpopulations were present in periodontium. When a panel of aGPCR genes was projected onto the UMAP atlas of periodontium (Fig. 1b), the data showed that CD97 (*Adgre5*) highly expressed in monocyte/macrophage clusters. Additionally, cells from alveolar bone were divided into 12 clusters: neutrophil—*Ly6g*, myeloid progenitor—*Mpo*, macrophage—*Cd68*, B lymphocyte—*Cd79a*, pre-B lymphocyte—*Vpreb1*, haematopoietic stem and progenitor cell (HSPC)—*c-Kit*, dendritic cell (DC)—*Siglech*, erythroid cell—*Hbb-bt*, T lymphocyte—*Cd3g*, natural killer cell (NK)—*Klrd1*, megakaryocyte—*Ms4a2* and mesenchymal cell—*Col1a1* (Fig. 1c and Supplementary Fig. 1b). Similar to the above results, macrophage subpopulations were also present in the alveolar bone and CD97 (*Adgre5*) was highly expressed in macrophage clusters (Fig. 1d).

### Static compression upregulates CD97 expression in monocytes-macrophages and suppressed osteoclastogenesis

**Fig. 2:**
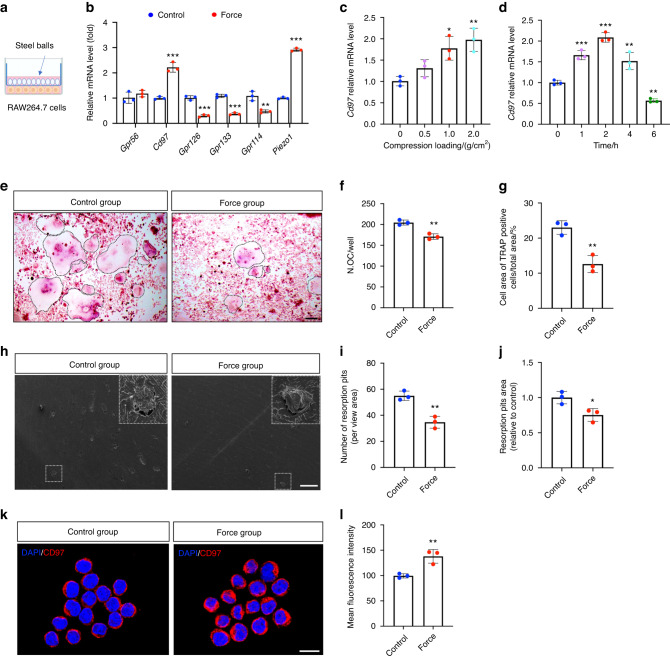
Static compression upregulates CD97 expression in monocytes-macrophages and suppressed osteoclastogenesis. **a** Schematic illustration of applying the compression. **b** Quantitative real-time polymerase chain reaction (qRT‒PCR) analysis of the expression of adhesion G protein-coupled (aGPCR) in RAW264.7 cells under static compression. **c** qRT‒PCR analysis of the expression of *Cd97* in RAW264.7 cells under different compression loadings. **d** qRT‒PCR analysis of the expression of *Cd97* in RAW264.7 cells under different loading times. **e** Tartrate-resistant acid phosphatase (TRAP) staining of osteoclasts, which were treated with 1 g/cm^2^ static stress for 4 h and induced with 50 ng/mL receptor activator of nuclear factor kappa-Β ligand (RANKL) for 7 days. The osteoclasts are highlighted by black dotted lines. Scale bar, 100 μm. **f** Quantification of osteoclast numbers with or without static stress stimulation (*n* = 3). **g** Percentage of cell area of TRAP-positive cells accounting for total observation area (*n* = 3). **h** Representative scanning electron microscopy (SEM) images of resorption lacunae on bovine bone slices with or without static stress stimulation. Large boxes show higher-magnification views of the small boxes. Scale bar, 500 μm. **i** Number of resorption pits per view area observed by SEM (*n* = 3). **j** Resorption pit area per view area observed by SEM (*n* = 3). **k** Fluorescence staining for CD97 after treatment with 1 g/cm^2^ static stress for 4 h. Scale bar, 10 μm. **l** Mean fluorescence intensity of CD97 on RAW264.7 with or without static stress stimulation (*n* = 3). Values are expressed as the mean ± standard deviation (SD). The *P* values were calculated by unpaired two-tailed Student’s *t* tests. *P < 0.05, **P < 0.01, ***P < 0.001

To investigate the effect of mechanical force on CD97 expression, we cultured RAW264.7 cells for 3 days and then subjected to static compression of 1 g/cm^2^ for 2 h (Fig. 2a). The mRNA expression of *Piezo1* was upregulated, indicating that compression was successfully applied (Fig. 2b).^24^ We found that *Cd97* mRNA expression was upregulated compared to that in the control group, while that of other members, such as *Gpr126*, *Gpr133* and *Gpr114* were downregulated.

Furthermore, we detected the mRNA expression level of *Cd97* under different forces and times. *Cd97* mRNA expression was upregulated in a force-dependent manner and increased under both 1 and 2 g/cm^2^ stimulation for 2 h (Fig. 2c). We also found that static compression application significantly enhanced *Cd97* mRNA expression during the early stages of stimulation (1, 2 and 4 h; Fig. 2d). The optimal compression condition of 1 g/cm^2^ for 4 h was identified by staining for viability (Supplementary Fig. 2). Tartrate-resistant acid phosphatase (TRAP) staining was used to observe and evaluate osteoclastogenesis, and positive cells were defined as cells containing at least three nuclei (Fig. 2e). The number of osteoclasts (Fig. 2f) and percentage of the area of TRAP-positive cells (Fig. 2g) significantly decreased after compression loading and RANKL-induced osteoclastogenesis for 7 days.

To assess the bone resorption function of osteoclast, we seeded RAW264.7 cells on dentin slices and observed by scanning electron microscopy (SEM) (Fig. 2h). The number of resorption pit formation (Fig. 2i) and resorption pit area (Fig. 2j) decreased significantly, compared to those of the control group. In addition, immunofluorescence staining confirmed that compression loading upregulated CD97 expression following static compression (Fig. 2k, l).

### CD97 expression increases in the macrophages of periodontal tissue during early orthodontic force loading

**Fig. 3:**
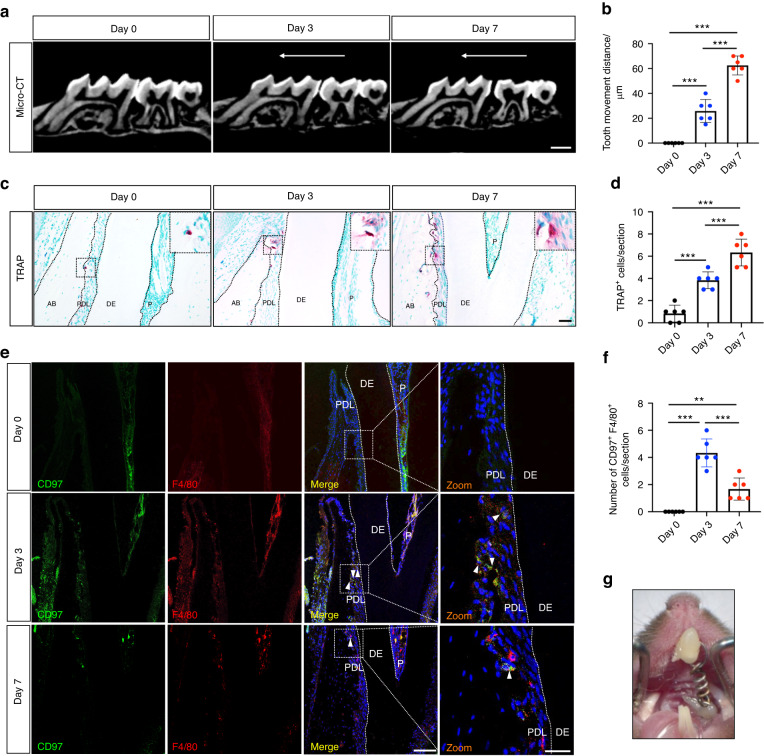
CD97 expression increases in the macrophages of periodontal tissue during early orthodontic force loading. **a** Representative microcomputed tomography (micro-CT) images following orthodontic tooth movement (OTM) for 0 d, 3 d and 7 d. The white arrow shows the direction of OTM. Scale bar, 500 μm. **b** Quantitative micro-CT analyses of the tooth movement distance between the first and second molars (*n* = 6). **c** Representative tartrate-resistant acid phosphatase (TRAP)-positive cells in the periodontal tissue of the mesiobuccal root of the upper first molar. Large boxes show higher-magnification views of the small boxes. AB alveolar bone, PDL periodontal ligament, DE dentin, P pulp. Scale bar, 50 μm. **d** Statistics of the numbers of TRAP-positive cells (*n* = 6). **e** Immunostaining of CD97^+^ and F4/80^+^ cells in the periodontal tissue after 0 d, 3 d and 7 d of OTM. Green shows the signal of CD97 staining; red shows the signal of F4/80; blue shows DAPI staining. Positive cells are indicated by white tringles. PDL, periodontal ligament; DE, dentin; P, pulp. Scale bar with low magnification, 100 μm; Scale bar with high magnification, 25 μm. **f** Quantification of the numbers of CD97^+^ and F4/80^+^ cells per section (*n* = 6). **g** Representative image of the application of orthodontic force to the first molar. Values are expressed as the mean ± SD. The *P* values were calculated by one-way analysis of variance with Tukey’s post hoc test. *P < 0.05, **P < 0.01, ***P < 0.001

To explore the expression of CD97 in the macrophages of periodontal tissue under orthodontic compression, we established an OTM model (Fig. 3g) and collected the maxillae on days 0, 3, and 7. Microcomputed tomography (micro-CT) was performed to evaluate the distance moved by the maxillary first molar. The OTM distance increased significantly on day 7 compared to that on day 3 (Fig. 3a, b). We found that the number of osteoclasts increased from day 0 to 7 through TRAP staining (Fig. 3c, d). The expressions of F4/80 were examined in periodontal tissue as F4/80 antigen is macrophage marker in mice.^25^ Immunofluorescent colocalization for CD97^+^ F4/80^+^ macrophages showed more positive cells in the periodontal tissue on day 3. Moreover, the number of CD97^+^ F4/80^+^ macrophages gradually decreased, which was negatively associated with the OTM distance (Fig. 3e, f).

### CD97 mediates mechanotransduction in osteoclast differentiation

**Fig. 4:**
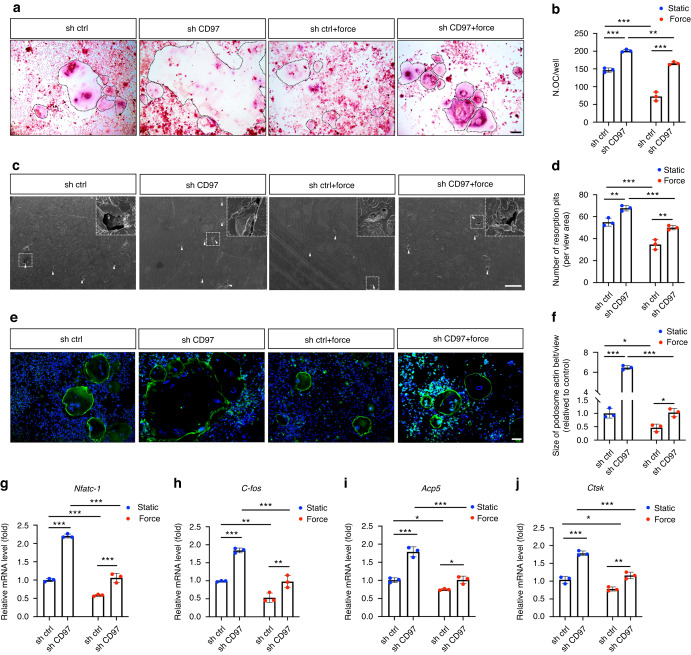
CD97 mediates mechanotransduction in osteoclast differentiation. **a** Representative tartrate-resistant acid phosphatase (TRAP) staining of osteoclasts in control or CD97 knockdown RAW264.7 cells cultured with or without static compression. The osteoclasts are highlighted by black dotted lines. Scale bar, 100 μm. **b** Quantification of the number of osteoclasts. **c** Representative scanning electron microscopy (SEM) images of resorption lacunae on bovine bone slices in control or CD97 knockdown RAW264.7 cells cultured with or without static compression. Resorption lacunae are indicated by white tringles. The white box area indicates a higher magnification. Scale bar, 500 μm. **d** Quantification of the number of resorption pits. **e** Representative podosome actin belt of osteoclasts with fluorescein isothiocyanate (FITC)-phalloidin staining and nuclei stained with DAPI. Scale bar, 100 μm. **f** Quantitative analysis of podosome actin belt size. **g**–**j** Quantitative real-time polymerase chain reaction (qRT‒PCR) analysis of the osteoclast differentiation-related genes in control or CD97 knockdown RAW264.7 cells cultured with or without static compression. Values are expressed as the mean ± SD. *P* values were calculated using two-way analysis of variance (ANOVA) with Tukey’s post hoc test. *P < 0.05, **P < 0.01, ***P < 0.001

To identify the function of CD97 in osteoclast formation, we knocked down *Cd97*/ *Adgre5* mRNA in RAW264.7 cells by lentivirus carrying different short hairpin RNAs (shRNAs) (Supplementary Fig. 3a). The knockdown efficiency of *Cd97*/*Adgre5*-specific shRNA was 60%–70% (LV-*Adgre5*-sh1), 50% (LV-*Adgre5*-sh2) and 40% (LV-*Adgre5*-sh3) (Supplementary Fig. 3b–d). In this notion, we chose LV-Adgre5-sh1 for the following experiment. TRAP staining demonstrated that the knockdown of CD97 stimulated osteoclast differentiation (Fig. 4a, b). CD97 deficiency promoted resorption pit formation (Fig. 4c, d). The size and number of podosome actin belt-positive osteoclasts increased in the CD97 knockdown group (Fig. 4e, f). In addition, knockdown of CD97 upregulated the expression of genes related to osteoclast differentiation (Fig. 4g–j).

To determine whether CD97 in monocytes-macrophages is essential for sensing mechanical loading, RAW264.7 cells were exposed to static compression following CD97 knockdown; 1 g/cm^2^ static compression loading for 4 h inhibited osteoclast formation and resorption. Knockdown of CD97 partially rescued osteoclastogenesis under compression (Fig. 4a, b). Consistently, the osteoclast resorptive function (Fig. 4c–f) and osteoclast differentiation-related genes were rescued (Fig. 4g–j). These results suggested that CD97 may serve as an important mechanosensitive receptor during osteoclast differentiation.

### The Rap1a/ERK signalling pathway mediates the effects of CD97 on osteoclast differentiation under compression stimulation

**Fig. 5:**
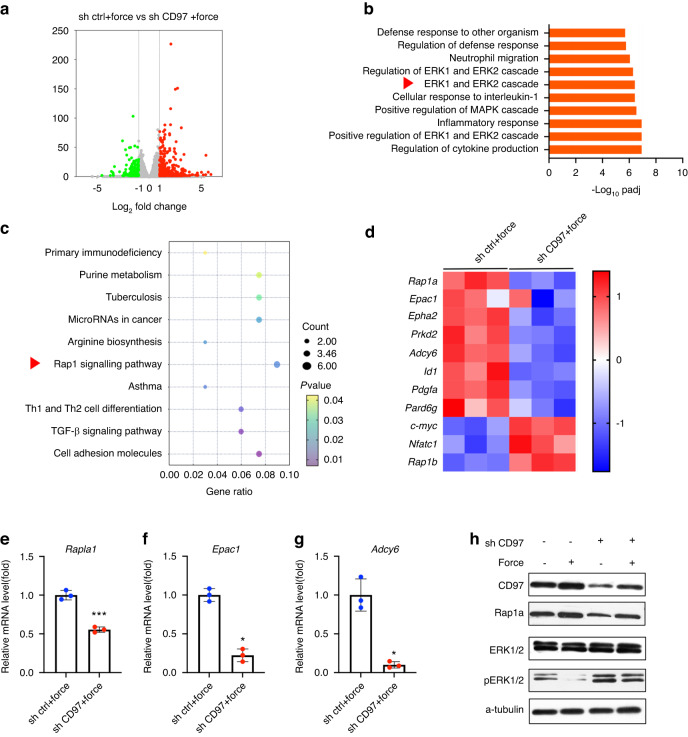
The Rap1a/ERK signalling pathway mediates the effects of CD97 on osteoclast differentiation under compression stimulation. **a** Volcano map of differentially expressed genes between the control group (sh ctrl) and CD97 knockdown group (sh CD97) under compression. **b** Gene Ontology (GO) enrichment analysis of the ten most significant terms in the biological processes. **c** Kyoto Encyclopedia of Genes and Genomes (KEGG) pathway analysis of the downregulated genes. The Rap1 pathway was the top downregulated pathway. **d** Heatmap of representative genes associated with Rap1 signalling. **e**–**g** Quantitative real-time polymerase chain reaction (qRT‒PCR) analysis of the mRNA levels of the Rap1 pathway. **h** Western blot analysis of the Rap1 signalling. Values are expressed as the mean ± SD. The *P* values were calculated by unpaired two-tailed Student’s *t* tests. *P < 0.05, **P < 0.01, ***P < 0.001

To determine the mechanism by which CD97 inhibits osteoclast differentiation under compressive loading, we performed RNA sequencing analysis (RNA-seq) of RAW264.7 cells transfected with negative control shRNA (LV-shNC) and CD97 shRNA (LV-shCD97). The cells were incubated for 3 days and loaded with compression stress for 4 h. The results demonstrated that 396 genes were upregulated and 176 were downregulated (Fig. 5a). The analysis of Gene Ontology (GO) revealed that the upregulated gene set was enriched in the ERK1 and ERK2 cascades (Fig. 5b). Kyoto Encyclopedia of Genes and Genomes (KEGG) analysis showed that the Rap1 signalling pathway was inhibited in RAW264.7 cells with CD97 knockdown under compression (Fig. 5c). Heatmap depicted the regulation of genes of interest related to the Rap1 pathway. Notably, the expression of Rap1a was down-regulated in CD97 knockdown group under compression (Fig. 5d). Congruent with our RNA-seq data, qRT‒PCR confirmed that CD97 knockdown downregulated the expression of representative genes of the Rap1 signalling pathway, such as *Rap1a*, *Epac1* and *Adcy6* (Fig. 5e–g). Because Rap1 activation plays an important role in regulating cellular functions by inhibiting the ERK pathway, which is essential for osteoclast differentiation, we also examined the protein levels of Rap1a and ERK in macrophages. CD97 knockdown downregulated the level of Rap1a and upregulated the phosphorylated ERK expression. Under compression stimulation, the protein level of CD97 and Rap1a increased, and the phosphorylated ERK expression decreased. Knockdown of CD97 partially rescued the expression of phosphorylated ERK under compression (Fig. 5h).

### Mechanical force regulates osteoclast differentiation and OTM via Rap1a/ERK signalling

**Fig. 6:**
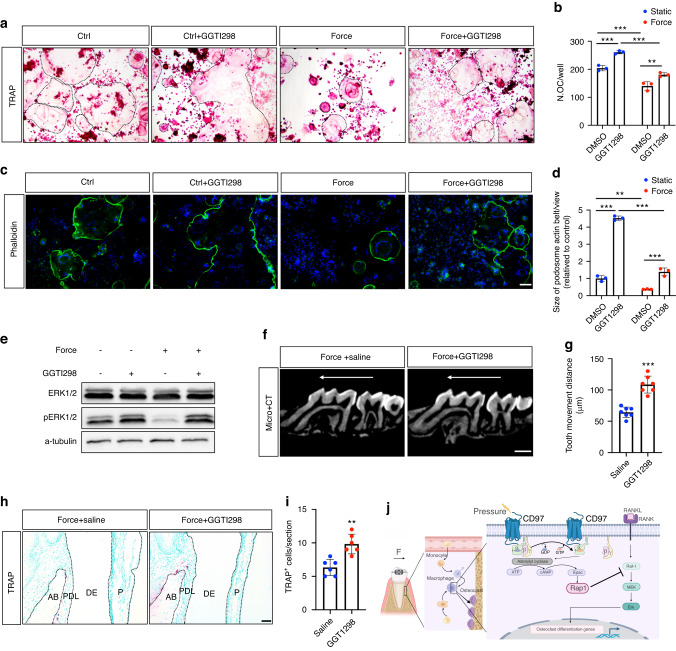
Mechanical force regulates osteoclast differentiation and orthodontic tooth movement (OTM) via Rap1a/ERK signalling. **a** Representative tartrate-resistant acid phosphatase (TRAP) staining of osteoclasts after static or compression stress loading with or without GGTI298. The osteoclasts are highlighted by black dotted lines. Scale bar, 100 μm. **b** Quantification of the osteoclast numbers. **c** Representative podosome actin belt of osteoclasts after static or compression stress loading with or without GGTI298. Scale bar, 100 μm. **d** Quantitative analysis of the size of the podosome actin belt. **e** Western blot analysis of the of ERK signalling. **f** Microcomputed tomography (micro-CT) images following OTM for 7 d with GGTI298 injection. The white arrow shows the direction of OTM. Scale bar, 500 μm. **g** Quantitative micro-CT analyses of the tooth movement distance with GGTI298 injection (*n* = 6). **h** Representative TRAP staining of osteoclasts in periodontal tissue after OTM with GGTI298 injection. Scale bar, 50 μm. **i** Statistics of the numbers of TRAP-positive cells (*n* = 6). **j** Schematic overview of the mechanisms by which CD97 suppresses osteoclast differentiation via the Rap1a/ERK pathway under compression

To determine whether Rap1a/ERK signalling is involved in osteoclast differentiation under mechanical force stimulation, we performed the Rap1a inhibitor GGTI298 with static compression in RAW264.7 cells. We found that static compression inhibited osteoclast formation and activity through TRAP staining and Phalloidin-FITC staining. GGTI298 rescued osteoclast differentiation and activity under compression (Fig. 6a–d). Western blotting revealed that phosphorylated ERK expression was downregulated by compression, and which was partially reversed after treatment with GGTI298 (Fig. 6e).

Next, we administered GGTI298 to mice in the unilateral first molar during OTM. Consistently, micro-CT analysis revealed that GGTI298 injection accelerated tooth movement (Fig. 6f, g) and that TRAP-positive cells increased in the periodontal ligament compared to those of the control group (Fig. 6h, i). Taken together, these results revealed that Rap1a suppression stimulates osteoclast differentiation and accelerates OTM.

## Discussion

Here, we revealed that CD97, a novel mechanosensitive aGPCR, enables macrophages to sense compression and integrate this information with Rap1a/ERK signals to inhibit osteoclastogenesis and OTM. Specifically, CD97 is expressed in mononuclear macrophages in the alveolar bone and periodontium. Compression upregulated CD97 expression and inhibited osteoclast differentiation. In addition, CD97-mediated mechanosensation and its downstream Rap1a/ERK signalling pathway are essential for modulating osteoclast differentiation and impedes early tooth movement. This understanding of how compression affects osteoclast differentiation may lead to novel mechanistic insights into the biological responses of the periodontium in OTM and, ultimately, to new strategies for orthodontic treatment.

OTM is a dynamic balance between alveolar bone resorption and formation. Osteoclast-mediated bone resorption influences the rate of OTM.^26^ At the early stage of OTM, macrophages accumulate on the compressive side of the periodontal tissue. Indeed, monocytes/macrophages have been proven to be osteoclast precursors and participate in bone resorption following differentiation.^27^ Previous studies have suggested that orthodontic compression stimulates periodontal fibroblasts and osteocytes to release a series of cytokines via mechanosensitive receptors. These cytokines indirectly regulate osteoclastic differentiation.^28,29^ Recent research has shown that macrophages are sensitive to mechanical stimuli through mechanosensitive receptors and are involved in osteoclastogenesis.^30^ Interestingly, the effect of compression on osteoclast differentiation remains controversial. Compression has been reported to induce osteoclastogenesis by upregulating the expression of osteoclast differentiation genes.^31,32^ However, other studies have shown that compression suppresses osteoclastogenesis,^33,34^ which is consistent with our observations. The contradictory conclusions may be linked to the different observation time points, loading pressures and force application systems.

As compressive stress directly influences osteoclast differentiation, it is essential to thoroughly investigate how macrophages perceive mechanical force to regulate osteoclastogenesis. aGPCRs are a subgroup of the GPCR superfamily and are closely related to mechanosensing.^35,36^ We demonstrated that CD97, a novel mechanosensitive aGPCR, is expressed in mononuclear macrophages in the alveolar bone and periodontium. Mechanical stimulation upregulated the expression of CD97 in macrophages and inhibited osteoclastogenesis. Conversely, knockdown of CD97 stimulated osteoclast differentiation, suggesting that CD97 is probably involved in bone remodelling during OTM. However, a few studies have shown that CD97 negatively regulates osteoclast differentiation, which diverges from the outcomes presented in our current work.^37,38^ The opposite results may be related to different cells used for inducing osteoclast differentiation, hormonal influences in vivo, or the encoding gene being stably knocked down or mutated.

The mechanical force sensing function of CD97 has recently attracted the attention of researchers.^23,39^ Our work highlights that CD97 is an important mechanoreceptor in macrophages and is involved in the regulation of osteoclast differentiation under stress. The results revealed that the force-induced expression of CD97 negatively regulated osteoclast differentiation, which was rescued by knockdown of CD97 with siRNA. To determine whether stresses are indeed loaded onto macrophages, we observed the expression of Piezo1 at the same time. Piezo1 is an important mediator of mechanotransduction.^40^ We found that the Piezo1 mRNA level was increased, which was consistent with the changes in CD97. However, Piezo1 deficiency in osteoclasts did not affect bone mass.^41^ It is suggested that CD97 sensing mechanical force is important in regulating osteoclast differentiation.

Mechanistically, KEGG analysis confirmed that Rap1 signalling plays an important role during osteoclast differentiation. Rap1 is a small G protein in the Ras superfamily, which is an important “molecular switch” in organisms.^42^ Rap1 has two isoforms: Rap1a and Rap1b. Upon activation, Rap1 triggers downstream signalling pathways.^43^ Although Rap1 has been reported to be involved in the regulation of bone resorption,^44^ whether the two isomers play the same role has not been elucidated. Rap1b overexpression increased TRAP-positive multinucleated cells, and Rap1b inhibition decreased the osteoclast numbers.^45^ In male Caucasians with discordant hip bone mineral density, Rap1b were identified based on multiple omics evidence. This suggests that Rap1b may be a critical factor in bone resorption.^46^ However, the relationship between Rap1a and osteoclastogenesis is not clear. We found that the expression of Rap1a downregulated and Rap1b upregulated in shCD97 group under compression. Therefore, we believe that the two isoforms of Rap1 play different role in osteoclastogenesis. As KEGG analysis showed that the Rap1 signalling pathway was inhibited in RAW264.7 cells with CD97 knockdown under compression, we finally selected Rap1a for the following mechanism studies.

GO analysis revealed that the upregulated genes were enriched in the ERK pathway following the knockdown of CD97 mRNA. As the ERK pathway plays an important role in modulating osteoclastogenesis and locates downstream of Rap1,^13^ we speculate that CD97 negatively regulates differentiation and activation of osteoclasts through Rap1a/ ERK pathway. Notably, a previous study demonstrated that deletion of both Rap1a and Rap1b in vitro resulted in osteoclasts with defective cytoskeletal organization and bone resorption capacity.^47^ However, we cannot conclude from this study that the two Rap1 isoforms play the same role in osteoclastogenesis. As Rap1b may play a predominant role in the above process. In addition, the effect of Rap1a on osteoclast differentiation was not demonstrated. Furthermore, we observed the expression of *Adcy6* and *Epac1*, downstream of CD97, decreased in shCD97 group under compression. Adenylyl cyclase can increase the concentration of intracellular cAMP, which combines with Epac1.^48,49^ Therefore, our data provides evidence that CD97 stimulates Adcy6 and Epac1 under mechanical stress, ultimately activates Rap1 signalling and regulates ERK signalling.

Moreover, to verify whether Rap1a/ERK signalling is involved in CD97-mediated mechanical force transduction, we used the GGTI298 in combination with compression in vitro and in vivo. GGTI298 is a CAAZ peptidomimetic geranylgeranyltransferase I (GGTase I) inhibitor, strongly inhibiting the processing of geranylgeranylated Rap1a.^50^ We found that GGTI298 reversed the inhibition of osteoclast differentiation induced by compression in vitro and accelerated OTM in vivo. These results suggest that mechanosensitive CD97 suppresses osteoclast differentiation via the Rap1a/ERK pathway under compression. We believe that our findings will be useful to all clinicians, especially orthodontists and researchers studying methods to accelerate OTM.

The current study has several limitations. First, there are some differences between humans and mice at both the genetic and epigenetic levels. Hence, relevant clinical samples should be collected to further elucidate the function of CD97 in OTM. Second, whether different types of mechanical stimulation affect CD97 expression should be explored in detail in the future. In addition, we will generate macrophage-specific CD97 knockout mice in the OTM model, which could make our results more reliable and persuasive. Moreover, the mechanism by which CD97 modulates Rap1 under mechanical force stimulation needs to be clarified in the future.

In conclusion, our results indicate that CD97 suppresses osteoclast differentiation through the Rap1a/ERK signalling pathway under orthodontic compression (Fig. 6j). These findings provide a potential target for accelerating OTM in the future.

## Materials and methods

### Cell culture

The murine macrophage line RAW264.7 (National Collection of Authenticated Cell Cultures) was cultured in Dulbecco’s modified Eagle’s medium (DMEM, Gibco, #C11995500BT) with 10% foetal bovine serum (Gibco, #10099-141) and 1% (v/v) penicillin/streptomycin (Gibco, #15140-122). Cells were stored at 37 °C in a moist atmosphere with 5% CO_2_. For osteoclast differentiation, RAW264.7 cells were transferred to 12-well plates at a density of 3 × 10^4^ cells per well, and 50 /mL soluble RANKL (Amizona, #AM1004) was added. The medium was changed every 2 days. In addition, 10 μmol/L GGTI-298 (Cayman, #16176) was used to treat RAW264.7 cells in the following experiment.

### Static compression application

RAW264.7 cells were cultured for 3 days and then compressed continuously by a force device as previously described.^51^ The principle of the compression device is illustrated in Fig. 2. The compression was changed by adjusting the number of steel balls in the container. RAW264.7 cells were subjected to 0.5, 1.0 or 2.0 g/cm^2^ compression for 1, 2, 4 or 6 h. As a control, cells were maintained without a force device.

### Live/dead viability staining

RAW264.7 cells (3 × 10^4^ cells per well) were seeded in 12-well plates for 72 h and subjected to 0.5, 1.0 or 2.0 g/cm^2^ compression for 1, 2, 4 or 6 h. A calcein/PI cell viability/cytotoxicity assay kit (Beyotime, #C2015S) was used for fluorescence detection. After washes with PBS solution, 500 μL of calcein AM/PI was added to the plate for 30 min in the dark. The living (green) and dead (red) cells were observed under a fluorescence microscope (Olympus, IX73). As cells had optimal viability at 1 g/cm^2^ of compression for 4 h, we finally chose the above conditions for subsequent experiments.

### TRAP staining

TRAP staining was used to observe and evaluate osteoclastogenesis in vivo and in vitro. For TRAP staining in vitro, cells were washed with PBS three times and fixed with 4% paraformaldehyde (PFA, Biosharp, #BL539A) for 30 min. Then, the cells were stained with TRAP staining solution for 15 min at 37 °C, which contained 50 mmol/L sodium tartrate (Sigma-Aldrich, #BCBS5706), 0.1 mol/L sodium acetate buffer (Sigma-Aldrich, #SLCH4856), napthol AS-MX phosphate (Sigma-Aldrich, #SLBV4822), and Fast Red Violet LB salt (Aladdin, #H2110208).

For TRAP staining in vivo, mandible samples were fixed in 4% PFA at 4 °C for 24 h, followed by decalcification with 10% EDTA for 6 weeks and embedding in paraffin. Cross sections were dissected at 5 μm and stained with TRAP staining for 15 min. Methyl green (Sigma, #MKBX8539V) was used to restain the sections for 15 s. The number of osteoclasts was counted in the periodontal tissue of the mesiobuccal root of the upper first molar.

### Bone resorption experiments

The bovine bone slices were prepared as previously described.^52^ RAW264.7 cells were cultured on bone slices in 12-well plates (3 × 10^4^ cells per well). The medium was changed every 2 days. After 7 days of culture, the bone slices were harvested. Then, sonication was used to remove the cells. Samples were then fixed in 4% PFA for 15 min and treated in 0.3% H_2_O_2_ (Boster, #16C01A) for 30 min. Slices were dried and coated with gold for SEM detection and viewed at 20 kV. Resorption pit area measurements were performed using ImageJ v2.9.0 software (National Institute of Health). Three views were randomly selected from each slice to examine the number of pits and the resorption pit area.

### Immunofluorescence staining

RAW264.7 cells were seeded on a glass coverslip in 12-well plates (5 × 10^4^ cells per well). On day 3, the cells were subjected to 1 g/cm^2^ compression for 4 h and then fixed in 4% PFA for 20 min. After three washes with PBS, the cells were permeabilized with 0.5% Triton X-100 (Sigma, #SLCD3084) for 30 min and then incubated in blocking buffer (1% BSA, 5% goat serum, and 0.1% Triton X-100) for 1 h. The cells were stained with diluted mouse anti-CD97 (Proteintech, #66972-1-Ig, 1:500) at 4 °C overnight. This step was followed by secondary antibody fluorescent labelling with goat anti-mouse Alexa Fluor 647 staining for 2 h at room temperature, and the nucleus was labelled with DAPI (Solarbio, #C0065) for 1 min. Images were captured by laser scanning confocal microscopy (Olympus, FV3000).

### Evaluation of podosome actin belt formation

After 7 days of osteoclast induction, RAW264.7 cells were fixed with 4% PFA at room temperature for 30 min and then permeabilized with 0.1% Triton X-100 (Sigma) in PBS for 15 min. Phalloidin-FITC (Solarbio, #CA1620) was used for podosome actin belt staining for 1.5 h, and then, the cells were counterstained with DAPI (Solarbio, #C0065) for 5 min in the dark. Samples were observed and imaged by inverted fluorescence microscopy (Olympus, IX73).

### Quantitative RT‒PCR

Cells were lysed with TRIzol (Invitrogen, #15596-026) for total RNA extraction. The concentration and purity of RNA were tested by a Nano300 microspectrophotometer (Thermo Fisher Scientific). Complementary DNA (cDNA) was synthesised using TaqMan Reverse Transcription Reagents (TaKaRa, #RR047A) according to the manufacturer’s instructions. Hieff qPCR SYBR Green Master Mix (Yeasen, #11201ES08) and gene-specific primers were used to perform qPCR as previously described.^53^ The primers used in this experiment are presented in Supplementary Table 1.

### Western blot

Western blot analysis was performed following standard procedures. In brief, cells were lysed in RIPA buffer (Sabbiotech, #PE001) with protease inhibitor and phosphatase inhibitor. Proteins were separated by SDS‒PAGE (Epizyme, #PG211, #PG212, #PG214) and transferred onto nitrocellulose membranes (PALL, #P-N66485). Then, the membrane was blocked with 5% w/v bovine serum albumin (BioFroxx, #143183) for 2 h and incubated with primary antibodies overnight at 4 °C, including CD97 (Proteintech, #66972-1-Ig, 1:500), Rap1a (Invitrogen, #MA1-013, 1:1 000), ERK1/2 (CST, #34370 S, 1:2 000), pERK1/2 (CST, #4695 S, 1:2 000) and a-tubulin (Proteintech, #11224-1, 1:5 000). The membranes were washed three times with TBST and incubated with the corresponding secondary antibodies (Signalway Antibody, #L3012-1, L3032-1, 1:10 000) for 1 h at room temperature.

### Lentivirus-mediated RNAi knockdown

RAW264.7 cells (5 × 10^4^ cells per mL) were cultured in 6-well plates. The next day, the cells were infected with lentivirus carrying three different shRNAs at a multiplicity of infection (MOI) of 25 (GeneChem). In addition, HitransG was added to enhance infection efficiency. The LV-Adgre5-sh1 targeting sequence was GCGTCTGTAACCTGGGATATA; the LV-Adgre5-sh2 targeting sequence was GAGTGTTTACTACCTGGATTT; and the LV-Adgre5-sh3 targeting sequence was CCATAGCACATGGCTATCCTA. RAW264.7 cells were infected with lentivirus for 72 h, and the knockdown efficiency was validated by RT‒qPCR and western blotting. For generation of a fully transduced population of cells, 4 μg/mL puromycin (Solarbio, #P8230) was used to eliminate nontransduced cells.

### RNA-seq

Total RNA from cells infected with lentivirus carrying shRNA and negative control was extracted using TRIzol (Invitrogen, #15596-026). An Agilent 2000 Bioanalyzer was utilised to detect the integrity and total amount of RNA, which was purified by magnetic beads enriched with poly-T oligos. Sequencing libraries were generated by the NEBNext ® Ultra^™^ RNA Library Prep Kit for Illumina® according to the manufacturer’s recommendations. After library quality assessment, different libraries were sequenced on an Illumina HiSeq 6000 machine, and 150 bp paired-end reads were generated. FastQC software was used to control the quality of raw reads and mapped to the *Mus musculus* genome using HISAT2 (v2.0.5). The differential expression of two conditions/groups was analysed by the DESeq2 R package (1.20.0). Genes were considered significantly differentially expressed if the fold change was ≥1.5 and the adjusted *P* value was <0.05. GO enrichment analysis of differentially expressed genes and KEGG pathway analysis were implemented by the clusterProfiler R package (3.8.1).

### scRNA-seq data analysis

Mouse alveolar bone scRNA-seq data were obtained from Sequence Read Archive (SRA) datasets (https://www.ncbi.nlm.nih.gov/sra/) via accession number PRJNA697839. The periodontal ligament scRNA-seq dataset (GSE160358) can be downloaded from the GEO database (https://www.ncbi.nlm.nih.gov/geo/). For the scRNA-seq analysis, the gene expression matrix of scRNA-seq data was downloaded and loaded into the Seurat package (v4.1). For quality control, cells with mitochondrial genes higher than 25% and fewer than 200 expressed genes were filtered out. Principal component analysis (PCA) was performed. The cells were divided into different subclusters with the FindClusters function at resolution = 1, and then, uniform manifold approximation and projection (UMAP) (dims = 1:30) was performed based on the PCA results to visualise the data. Cell types of different subclusters were annotated by specific marker genes using the FindVariableGens function. To determine the expression of aGPCR in various cellular components in the periodontium and alveolar bone, we selected 33 aGPCR genes for visualisation.

### Animal models

This study was approved by the Committee on Animal Research of Hebei Medical University (Approval number: 2023044). The ARRIVE (Animal Research: Reporting of In Vivo Experiments) guidelines were followed throughout the experiment. Male C57/BL6 mice (20–25 g, 8 weeks old) were purchased from Beijing HFK Bio-Technology. All animals were kept in the Hebei Key Laboratory of Stomatology at Hebei Medical University under standard pathogen-free conditions in a 12 h light-dark cycle. The OTM model was established as previously described.^54^ Briefly, mice were anaesthetised by pentobarbital sodium. The anaesthetic dose, administered intraperitoneally, was 40‒50 mg/kg body weight. For induction of OTM, 3 mm nickel-titanium spiral springs (IMD, #OS1218) were ligated between the left maxillary first molar and incisors and generated 10 g orthodontic force under activation. The contralateral side served as a control. Dental light curing flowable composite resin (SPIDENT, EsFlow A3) was used to prevent spring detachment. Animals were fed a soft diet to relieve discomfort. The appliances were assessed daily, and rebonding was performed if the spring fell off or was damaged. Six mice were sacrificed after application of orthodontic force for 0, 3, and 7 days. Tooth movement distance and histological evaluation was conducted in a single-blind manner.

### Injection of Rap1a inhibitor GGTI298

The orthodontic force was applied to both sides of the maxillary first molars and incisors. The mesial periodontium of the left side was injected with 25 μL of GGTI298 (Cayman, #16176; 50 μg/mL), and the right side was injected with an equivalent amount of PBS at the same site (*n* = 6). The rats were injected every 2 days during the experiments. The mice were sacrificed after the application of orthodontic force for 7 days.

### Micro-CT analysis

Mouse maxillae were harvested on day 0, day 3 and day 7 of the experiment and fixed in 4% PFA for 24 h. Samples were scanned by a Venus micro-CT system (PINGSHENG Healthcare) with a spatial resolution of 8 μm (90 kV, 65 μA). The three-dimensional structure was reconstructed by Avatar software. The tooth movement distance was measured in relation to the contact point of the distal-marginal ridge of the first maxillary molar and the mesial-marginal ridge of the second molar.^55^

### Histological immunofluorescence staining

Mandible samples including molars were fixed in 4% PFA at 4 °C for 24 h and decalcified with 10% EDTA for 6 weeks. Cross sections were dissected at 5 μm. Antigen retrieval was conducted in sodium citrate buffer (Solabio, #C1032) at 99 °C for 15 min. Then, the slides were incubated with diluted mouse anti-CD97 (Santa Cruz, #sc-166852, 1:500) and anti-F4/80 (Abcam, #ab6640) at 4 °C overnight. The sections were incubated with the specific secondary antibodies at room temperature and stained with DAPI for 5 min. The images were observed and captured under laser scanning confocal microscopy (Olympus, FV3000).

### Statistical analysis

All data are expressed as the mean ± SD. Unpaired two-tailed Student’s *t* tests were performed for comparisons between two groups. One-way ANOVA or two-way ANOVA followed by Tukey’s test for multiple comparisons was used to assess differences between groups. *P* < 0.05 was considered statistically significant. All statistical analyses were performed with Prism 9.0 (GraphPad, Inc.).

### Supplementary information

Supplementary materials

## Data Availability

The data supporting the findings are available from the corresponding author upon reasonable request.
