# Supplementary material for: CD97 inhibits osteoclast differentiation via Rap1a/ERK pathway under compression
Source: Int J Oral Sci. 2024 Feb 4;16:12. doi: 10.1038/s41368-023-00272-x (PMC10838930; doi:10.1038/s41368-023-00272-x)
Supplement: Supplementary file 1 — Supplementary materials [file 41368_2023_272_MOESM1_ESM.docx]

Supplementary information for

**CD97 inhibits osteoclast differentiation via Rap1a/ERK pathway under compression**

Wen Wang, Qian Wang, Shiying Sun, Pengfei Zhang, Yuyu Li, Weimin Lin, Qiwen Li, Xiao Zhang, Zhe Ma*, Haiyan Lu*

**Correspondence:*

1. Haiyan Lu

E-mai: [luhaiyan67@163.com](mailto:luhaiyan67@163.com)

Tel: +86 0311 86266590

2. Zhe Ma

E-mail: [mazhe@hebmu.edu.cn](mailto:mazhe@hebmu.edu.cn)

Tel: +86 0311 86261175

**This file includes:**

Supplementary Table: 1 table

Supplementary Figures: 3 figures

**Supplementary Table
Primers sequence used for qRT-PCR in this study**

| Genes | Forward / Reverse | Sequence (5' to 3') |
| --- | --- | --- |
| *Gpr56* | Forward | CAACTCAGACAGCGCCAAAC |
|  | Reverse | TGCTTCTGAGCCCTTTGAGG |
| *Gpr126* | Forward | TAGTTAGGCTTGGCAGTGGC |
|  | Reverse | CACTGGAACAGAACCCGTGA |
| *Cd97* | Forward | CAAGGCCTGAGTACATGGCA |
|  | Reverse | CAGCAGTATGTTGCATGCCC |
| *Gpr133* | Forward | GACCAGACCAAGCGTTACGA |
|  | Reverse | GCGTTTTCTCTCCTCGGTCA |
| *Gpr114* | Forward | CAAAGCGATTCCACCACACG |
|  | Reverse | AGTATCGGCGAATGTAGGCG |
| *Piezo1* | Forward | CTTACACGGTTGCTGGTTGG |
|  | Reverse | CACTTGATGAGGGCGGAAT |
| *Gapdh* | Forward | CATCACTGCCACCCAGAAGACTG |
|  | Reverse | ATGCCAGTGAGCTTCCCGTTCAG |
| *Nfatc1* | Forward | GGAGAGTCCGAGAATCGAGAT |
|  | Reverse | TTGCAGCTAGGAAGTACGTCT |
| *c-fos* | Forward | CGGGTTTCAACGCCGACTA |
|  | Reverse | TTGGCACTAGAGACGGACAGA |
| *Acp5* | Forward | CACTCCCACCCTGAGATTTGT |
|  | Reverse | CATCGTCTGCACGGTTCTG |
| *Ctsk* | Forward | GAAGAAGACTCACCAGAAGCAG |
|  | Reverse | TCCAGGTTATGGGCAGAGATT |
| *Rap1a* | Forward | AAAGGATCCTATGCGTGAGTACAAGCTAGTGGT |
|  | Reverse | AAAAGAATTCCTAGAGCAGCAGACATGATTTCT |
| *Epac1* | Forward | AAT​GGC​TGT​GGG​AAC​GTA​TCT​C |
|  | Reverse | CCT​GGT​TAG​GGA​GCC​AAA​CA |
| *Adcy6* | Forward | GCATCCTGTTTGCGGACATT |
|  | Reverse | ACAGTGATTCTCCCTCACCG |

**Supplementary Figures**

**
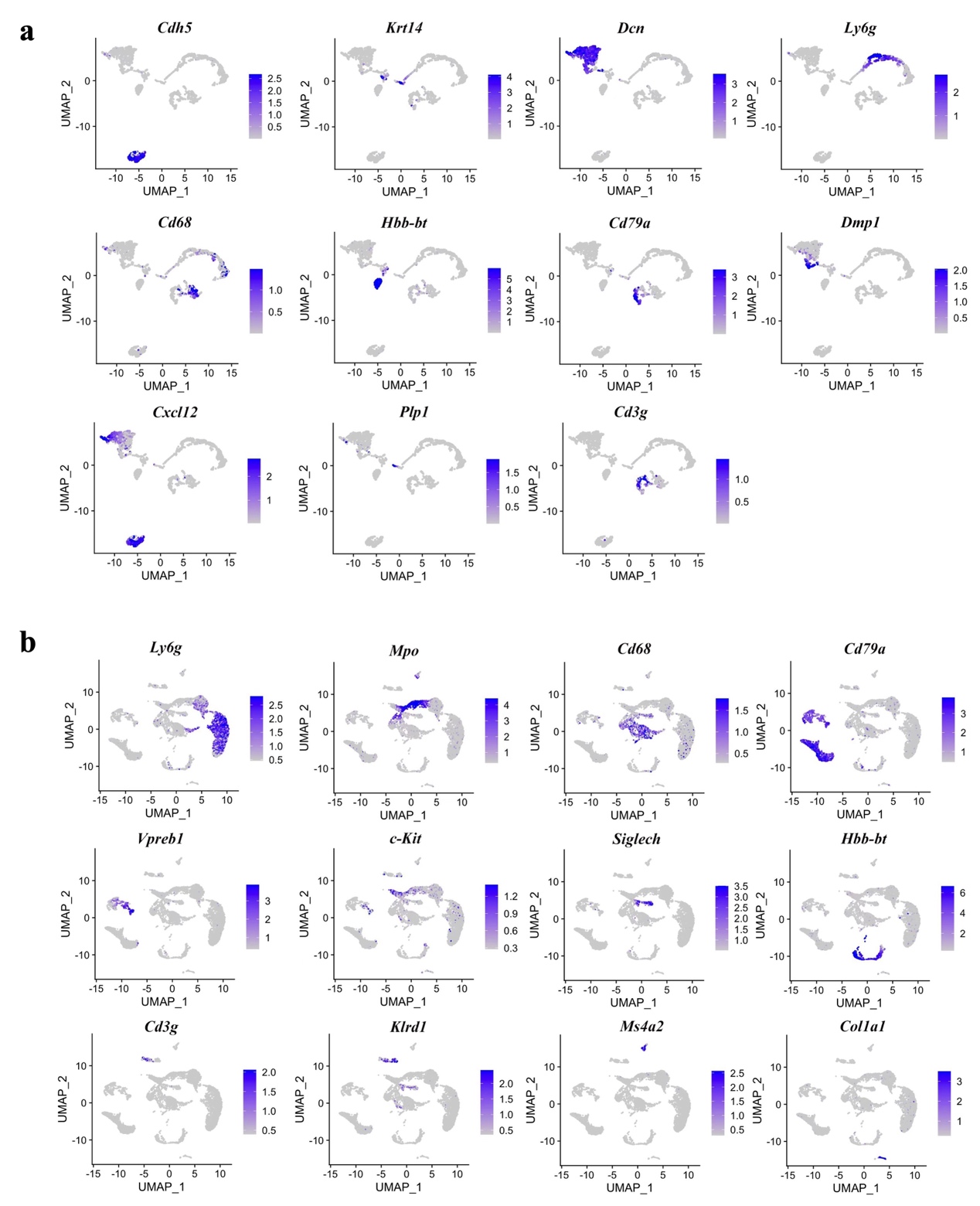
**

**Supplementary Figure 1. The expression patterns of classical marker genes in different cell types projected on the UMAP plot. a** Cells from periodontal tissue were identified into 11 clusters based on the classical cell marker genes. **b** Cells from alveolar bone were divided into 12 clusters according to the known marker genes.


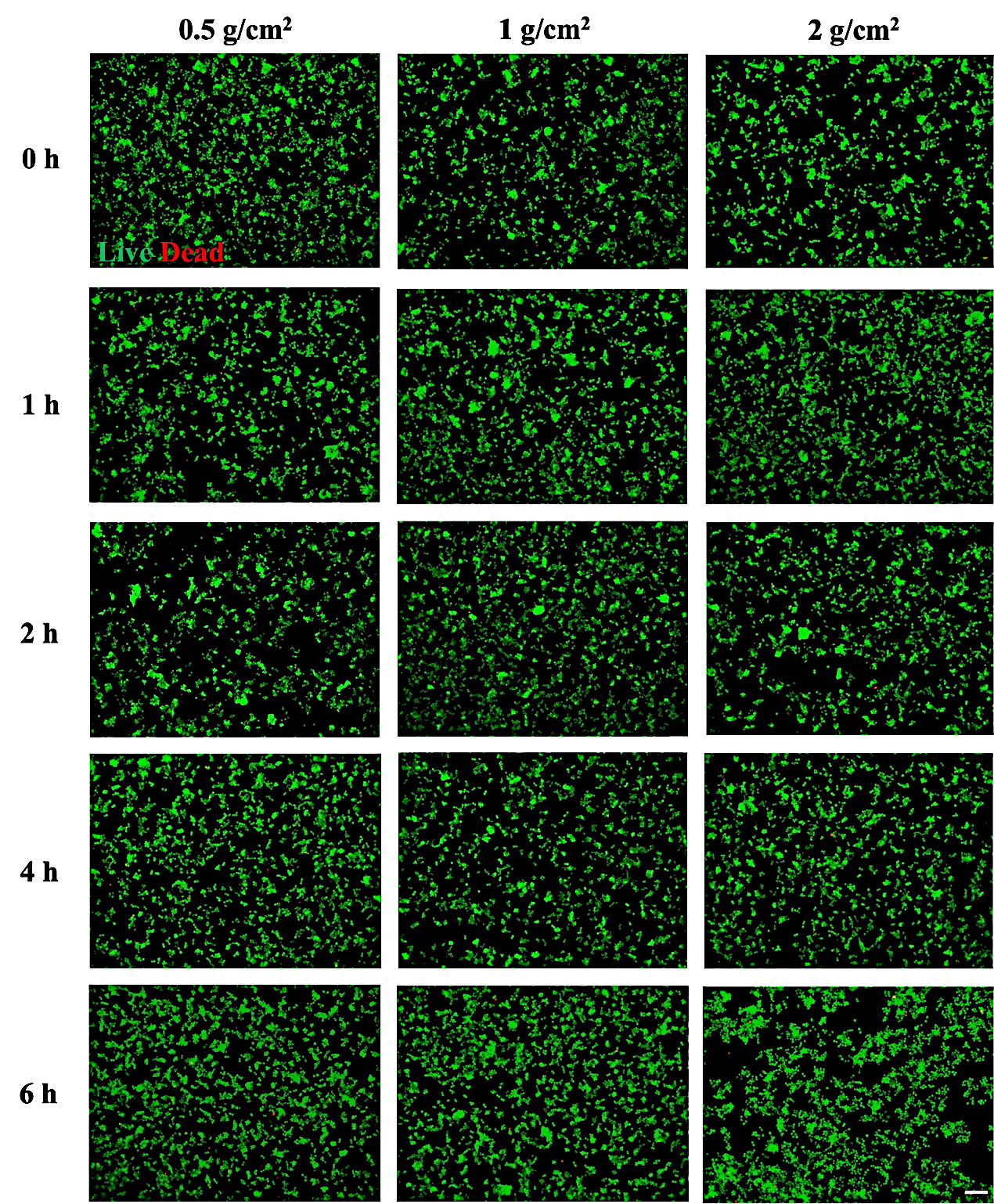


**Supplementary Figure 2. Live/Dead Viability Staining of RAW264.7 cells after mechanical compression.** The RAW264.7 cells were seeded in 12-well for 72h and subjected to 0.5 g/cm^2^, 1 g/cm^2^, 2 g/cm^2^ compression for 1h, 2 h, 4 h or 6 h. Cells were maintained under the uncompressed condition (0 h) as the control. Scale bar, 100 μm. The living cells were stained green, and the dead cells were stained red.

**
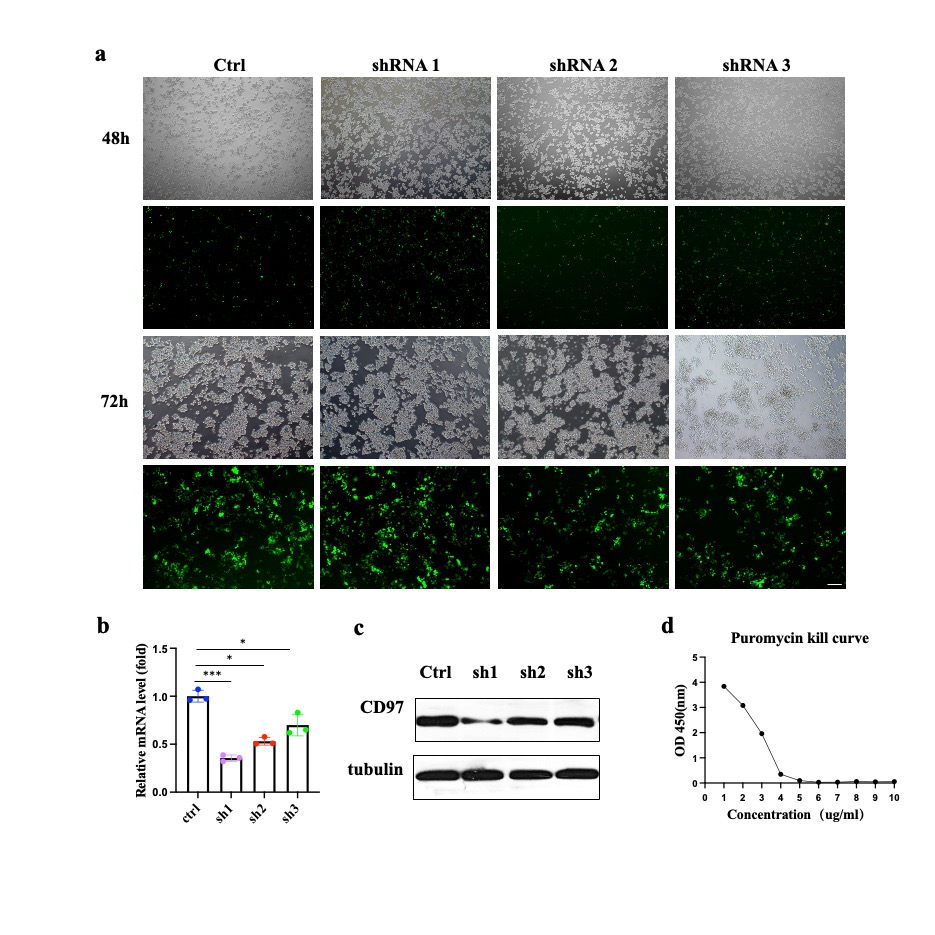
Supplementary Figure 3. Verification of transfection efficiency of CD97. a** Bright field and fluorescent microscopy images of RAW264.7 cells that were infected with lentivirus carrying different short hairpin RNA (shRNA). Scale bar, 100 μm. **b,c** qRT-PCR and Western blot confirmed successful knockdown of CD97 in RAW264.7 cells. **d** The Puromycin kill curve to determine the minimum amount to eliminate the non-transduced cells.
